# Metabolic stress-induced human beta-cell death is mediated by increased intracellular levels of adenosine

**DOI:** 10.3389/fendo.2023.1060675

**Published:** 2023-01-25

**Authors:** Anongnad Ngamjariyawat, Jing Cen, Romain Said, Ceren Incedal, Olof Idevall-Hagren, Nils Welsh

**Affiliations:** ^1^ Science for Life Laboratory, Department of Medical Cell Biology, Uppsala University, Uppsala, Sweden; ^2^ Division of Anatomy, Department of Preclinical Sciences, Faculty of Medicine, Thammasat University, Khlong Luang, Pathumthani, Thailand

**Keywords:** beta-cells, adenosine, apoptosis, PIP3K-signaling, metabolic stress, sodium palmitate

## Abstract

**Introduction:**

High intracellular concentrations of adenosine and 2’-deoxyadenosine have been suggested to be an important mediator of cell death. The aim of the present study was to characterize adenosine-induced death in insulin-producing beta-cells, at control and high glucose + palmitate-induced stress conditions.

**Methods:**

Human insulin-producing EndoC-betaH1 cells were treated with adenosine, 2’-deoxyadenosine, inosine and high glucose + sodium palmitate, and death rates using flow cytometry were studied.

**Results:**

We observed that adenosine and the non-receptor-activating analogue 2-deoxyadenosine, but not the adenosine deamination product inosine, promoted beta-cell apoptosis at concentrations exceeding maximal adenosine-receptor stimulating concentrations. Both adenosine and inosine were efficiently taken up by EndoC-betaH1 cells, and inosine counteracted the cell death promoting effect of adenosine by competing with adenosine for uptake. Both adenosine and 2’-deoxyadenosine promptly reduced insulin-stimulated production of plasma membrane PI(3,4,5)P_3_, an effect that was reversed upon wash out of adenosine. In line with this, adenosine, but not inosine, rapidly diminished Akt phosphorylation. Both pharmacological Bax inhibition and Akt activation blocked adenosine-induced beta-cell apoptosis, indicating that adenosine/2’-deoxyadenosine inhibits the PI3K/Akt/BAD anti-apoptotic pathway. High glucose + palmitate-induced cell death was paralleled by increased intracellular adenosine and inosine levels. Overexpression of adenosine deaminase-1 (ADA1) in EndoC-betaH1 cells, which increased Akt phosphorylation, prevented both adenosine-induced apoptosis and high glucose + palmitate-induced necrosis. ADA2 overexpression not only failed to protect against adenosine and high glucose + palmitate-activated cell death, but instead potentiated the apoptosis-stimulating effect of adenosine. In line with this, ADA1 overexpression increased inosine production from adenosine-exposed cells, whereas ADA2 did not. Knockdown of ADA1 resulted in increased cell death rates in response to both adenosine and high glucose + palmitate. Inhibition of miR-30e-3p binding to the ADA1 mRNA 3’-UTR promoted the opposite effects on cell death rates and reduced intracellular adenosine contents.

**Discussion:**

It is concluded that intracellular adenosine/2’-deoxyadenosine regulates negatively the PI3K pathway and is therefore an important mediator of beta-cell apoptosis. Adenosine levels are controlled, at least in part, by ADA1, and strategies to upregulate ADA1 activity, during conditions of metabolic stress, could be useful in attempts to preserve beta-cell mass in diabetes.

## Introduction

Adenosine is a key endogenous nucleoside composed of the purine adenine attached to a ribose. It is an important metabolite involved in different processes, such as energy storage, intermediary metabolism, gene expression, translation and cell-to-cell signalling (4). Intracellularly, *de novo* purine synthesis leads to the sequential formation of IMP, adenylosuccinate and AMP (5). AMP is then converted into adenosine by a 5’-nucleotidase that removes the 5’ phosphate group. Adenosine is often and rapidly phosphorylated back to AMP *via* the enzyme adenosine kinase. Adenosine (and inosine) can also be taken up from the extracellular space by nucleoside transporters, which are either equilibrative nucleoside transporters (ENTs) present in most cells, or concentrative nucleoside transporters (CNTs) present in epithelial cells (1, 2). If there is an increased need for ATP intracellularly, adenosine is rapidly phosphorylated by sequential steps. However, if levels of adenosine nucleotides must be reduced, AMP is deaminated to IMP by AMP deaminase (AMPD) or dephosphorylated to adenosine, which is then deaminated to inosine by the adenosine deaminase (ADA) enzymes ADA1 and ADA2 (2). Inosine is subsequently degraded intracellularly *via* deribosylation, executed by purine nucleoside phosphorylase (PNP), followed by conversion to uric acid. In addition, excess inosine and adenosine can be released to the extracellular space *via* ENT1/2 (2). 2’-deoxyadenosine, which is mainly utilized for the synthesis of DNA, is chemically quite similar to adenosine, and is transported and metabolized by the same transporters (CNTs and ENTs) and enzymes (AMPD, ADA and PNP) as adenosine (3).

Adenosine is not only a precursor for nucleotide synthesis, it functions also as a signaling molecule. There are four purinergic G-protein coupled receptors that are activated by adenosine: A_1_, A_2A_, A_2B_ and A_3_. Two of these are stimulatory (A_2A_ and A_2B_) and two are inhibitory (A_1_ and A_3_), and these receptors respond to adenosine concentrations in the approximate range between 0.01 − 15 μM. Inosine binding to these adenosine receptors is less potent and the ED_50_s for A_1_ and A_2B_ are as high as 290 μM and 89-301 μM, respectively (6). Adenosine-induced receptor activation promotes pleiotropic effects including changes in heart, lung, bone, neutrophil and synaptic function. Inosine-induced A_2B_ receptor activation induces anti-inflammatory effects (6). In pancreatic islets adenosine-induced signaling appears to stimulate insulin release and beta-cell regeneration (7).

Adenosine concentrations in the extracellular space are usually in the sub-micromolar range, but can reach as high as 50-100 μM during severe stress conditions. Intracellular adenosine concentrations, in cardiomyocytes, are normally in the nanomolar range, but can also increase considerably during different forms of stress (8). Adenosine concentrations in INS-1 beta-cells appear to be in the low μM range at basal conditions (9). Concentrations of inosine are generally higher than those of adenosine as inosine has a longer half-life (10).

Although adenosine toxicity on mammalian cell lines has been described as early as in the 70s (11), relatively few studies have focused on receptor-independent/intracellular mechanisms. Interest in the intracellular metabolism of purine analogues (adenosine and its derivative) arose after the understanding of the pathophysiology of a specific type of Severe Combined Immunodeficiency (ADA-SCID) found to be the result of an inherited deficiency in adenosine deaminase enzyme 1 (ADA1). In this disease there is an accumulation of intracellular adenosine and 2’-deoxyadenosine that is toxic for T-, B- and NK-lymphocytes (12). This has initiated an increasing interest in the intracellular actions of adenosine and 2’-deoxyadenosine that can be seen at higher (micro- to millimolar) concentrations. In fact, intracellular adenosine has been linked to increased hepatoma cell death, possibly *via* inhibition of autophagy (13). Beta-cell function may be negatively affected by high concentrations of adenosine *via* decreased autophagy (14). Inosine, on the other hand, appears to be associated with beneficial effects. Indeed, inosine has been suggested to function as a fuel in the glycolytic pathway (15), to promote anti-oxidative actions in neurons (16) and to stimulate survival of PC12 cells (17). It is also noteworthy that lowered expression of ADA1, which increases the adenosine/inosine ratio, resulted in increased death of motor neurons in amyotrophic lateral sclerosis (18). In NK cells high levels of inosine are beneficial whereas high levels of adenosine will lead to cytotoxicity and impaired innate immunity (19). Interestingly, an inosine analogue, INO-2002, has been reported to protect against diabetes in low-dose streptozotocin-induced and NOD mouse T1D-similar diabetes by restoring the beta-cell mass (20). Thus, it may be that the conversion of adenosine to inosine determines the survival of not only lymphocytes but also beta-cells, and that this could have important ramifications for the treatment of type 1 and 2 diabetes patients.

To our knowledge, the impact of intracellular purines for beta-cell survival has hitherto not been investigated. Therefore, the aim of the present study was to characterize adenosine-induced death in insulin-producing beta-cells, and the putative role of adenosine as an intracellular mediator of high glucose + sodium palmitate-induced beta-cell death. High glucose + palmitate is a culture condition that mimics the disturbed metabolism of type 2 diabetes *in vivo*, and it is known to promote disturbed mitochondrial function and beta-cell death *in vitro* (21).

## Methods

### Human EndoC-betaH1 cell culture

Human EndoC-betaH1 cells were grown on Geltrex Basement Membrane Matrix (Gibco)-coated culture vessels in DMEM/Ham’s F11 (50%/50%, vol/vol) with supplements as described previously (22). Cells were cultured at 37°C in humidified air containing 5% CO_2_.

### Human islets

Human islets were provided through the Uppsala facility for the isolation of human islets from Scandinavian brain-dead individuals. Human islets were cultured free-floating in Sterilin dishes in CMRL 1066 medium (ICN Biomedicals, Costa Mesa, CA, USA) containing 5.6 mmol/l glucose, 10% FCS and 2 mmol/l l-glutamine for 1–5 days prior addition of adenosine and inosine. Cell viability was assessed by PI and Hoechst staining followed by fluorescence microscopy.

### Evaluation of cell viability

EndoC-betaH1 (2×10^5^) cells were cultured in the presence of adenosine (Sigma), 2-deoxyadenosine (Sigma) or inosine (Sigma) at 5.6 glucose or in the presence of 22 mM glucose + 1.5 mM palmitate for 24 hours. A stock solution of sodium palmitate (100 mM) (Sigma Aldrich) in 50% ethanol was used for preparation of 1.5 mM palmitate in EndoC-betaH1 cell culture medium containing 2% fatty acid free BSA. Palmitate was allowed to complex with BSA at 37 °C for at least 30 min.

EndoC-betaH1 cells were also treated with different concentrations of the Bax inhibitor BAI1 (EMD Millipore Corp, 10 μM) and the PHLPP inhibitor/AKT activator NSC117097 (MedChemExpress) at culture conditions for 18 hours. After the different treatments, cells were trypsinized for 5 min, followed by labelling with propidium iodide (Sigma) (10 μg/ml), for 10 min. Then cells were measured for red fluorescence (FL-3) using flow cytometry (FacsCalibur, BD). In some experiments cells were stained for both Annexin V and PI using the Annexin-V-FLUOS Staining Kit (Roche) according to the instructions of the manufacturer. Data were acquired on a BD Accuri C6 plus flow cytometer with analysis of red fluorescence (FL-3, 650 nm) for PI and green fluorescence (FL-1, 530 nm) for Annexin V-FITC staining. PI positive cells (including both Annexin V positive and negative cells) and Annexin V positive cells (excluding PI positive cells) were gated separately, and were counted as necrotic/late apoptotic and early apoptotic, respectively.

### TIRF microscopy imaging of plasma membrane PIP3

The following plasmids were used: GFP_4_-GRP1_PH_ (PI[3,4,5]P_3_-selective biosensor (23)), mRFP-PH_Akt_ (PI[3,4]P_2_/PI[3,4,5]P_3_ biosensor), EGFP-C3 (used as a reference marker for mRFP-PH_Akt_) and mCherry-N1 (used as a reference marker for GFP_4_-GRP1_PH_). Transient transfection of EndoC-betaH1 cells grown on 25 mm coverslips was performed with 0.4 μg plasmid DNA and 0.5 μl Lipofectamine 2000 (Invitrogen) in 100 μl Opti-MEM containing 2.5x10^5^ cells for 4h followed by further culture in complete culture medium for 18-24h. EndoC-betaH1 cells were transferred to an extracellular-like buffer (125 mM NaCl, 4.8 mM KCl, 1.3 mM CaCl_2_, 1.2 mM MgCl_2_, 25 mM HEPES, 3 mM D-glucose, and 0.1% BSA [pH 7.40]) and incubated for 30 min at 37°C prior to imaging. The coverslip were thereafter used as exchangeable bottoms in an open Sykes-Moore chamber, placed on the stage of a fluorescence microscope, and subsequently perfused with the extracellular-like buffer at a rate of 0.3–0.5 ml/min supplemented at intervals with insulin (100 nM) (Sigma), adenosine (1 mM) and 2-deoxyadenosine (1 mM) at 37°C. TIRF microscopy was performed as previously described (23). Briefly, the TIRF microscope was built around an inverted Eclipse Ti-E microscope (Nikon) equipped with a TIRF illuminator and a 60×/1.45 NA plan-Apo objective (Nikon). Diode-pumped solid-state lasers (Cobolt, Hübner photonics, Solna, Sweden) provided 491-nm light for excitation of GFP and 561-nm light for mCherry/mRFP excitation. Emission light was separated by interference filters (527/27 for GFP, 587-LP for mCherry/,mRFP, both from Semrock, Rochester, NY) mounted in a filter wheel (Lambda 10-3, Sutter Instruments, Novato, CA) and detected with a back-illuminated DU-897 EMCCD camera (Andor Technology, Belfast, Northern Ireland) controlled by MetaFluor software (Molecular Devices). An electronic shutter (Sutter Instruments) prevented illumination of the samples between image captures. The ImageJ distribution Fiji (https://imagej.net/software/fiji/) was used for image analysis. Individual cells were manually identified, and regions of interests were drawn such that as much as possible of the cell footprint was contained within the region throughout the time course of the experiment. The fluorescence intensity from these regions was subsequently measured and the values obtained were background corrected. After normalizing the data to the prestimulatory level (F/F_0_) and the average values for the last two minutes of each stimulation were calculated and plotted. Statistical significance was assessed using the Student’s t-test.

### [3H]-adenosine and [3H]-inosine uptake into EndoC-betaH1 cells

The uptake of [2,8-^3^H]-adenosine (250 μCi/ml, ViTrax Radiochemicals, CA, USA) and [2,8-^3^H]-inosine (250 μCi/ml, ViTrax Radiochemicals, CA, USA) were used to measure nucleoside uptake. All experiments were carried out in Krebs-Ringer bicarbonate HEPES (KRBH) buffer with 10 mM glucose (pH 7.4). EndoC-betaH1 cells were cultured in 6-well plates in KRBH buffer containing 10 mM glucose in the absence or presence of 3 μM adenosine with 0.5 μCi [2,8-^3^H]-adenosine, 3 mM adenosine with 4.5 μCi [2,8-^3^H]-adenosine, 3 μM inosine with 0.5 μCi [2,8-^3^H]-inosine, or 3 mM inosine with 4.5 μCi [2,8-^3^H]-inosine for one minute and one hour at room temperature. The cells were then washed four times rapidly with cold phosphate-buffered saline (PBS) (pH 7.4), followed by solubilization for one hour in 1 ml of 5% Triton X-100 on ice. Radioactivity was measured by a liquid-scintillation spectrometer (Wallac System 1400; PerkinElmer). DPM values were converted to pmol adenosine/inosine using the specific activities of the nucleoside solutions.

### Measurement of mitochondrial function

Oxygen consumption rate (OCR) and extracellular acidification rate (ECAR) from EndoC-betaH1 cells were measured by Extracellular Flux Analyzer XF^e^96 (Seahorse Bioscience, MA, USA) as previously described (24). EndoC-betaH1 cells (3×10^4^ per well, 5 replicates for each condition) were seeded directly in Geltrex ECM Matrix-coated 96-well seahorse plate. After culture for 2 days, EndoC-betaH1 cells were pre-incubated with assay medium (Seahorse Bioscience) containing 2.8 mM or 20 mM glucose (pH adjusted to 7.4) for 60 min at 37 °C in air before the microplate was inserted into the Analyzer. During the measurement different concentrations of adenosine and inosine were injected after 20 min and measured for another 30 minutes. Then 2 μM each of oligomycin, FCCP, and the combination of rotenone and antimycin A were sequentially injected after certain time periods, to determine basal and maximal respiratory capacity of the cells. All OCR measurements were corrected for non-mitochondrial OCR. ECAR was measured in parallel.

### Immunoblot analysis

EndoC-betaH1 cells were washed with ice-cold PBS for 3 times, lysed in SDS sample buffer, boiled for 5 min and separated by SDS-PAGE. Proteins were electrophoretically transferred onto a Hybond-P membrane (GE Healthcare). Immunoblotting was performed by incubating with the following primary antibodies: rabbit anti-phospho-AMPKα (1:1000, Cell Signaling Technology Cat# 2531, RRID : AB_330330), mouse anti-SREBP1 (1:1000, Invitrogen), rabbit rabbit anti-phospho-GSK3α/β (1:1000, Cell Signaling Technology), rabbit anti-phospho-eIF2α (1:1000, Cell Signaling Technology), rabbit anti-phospho-PGC1α (1:1000, R&D), rabbit anti-phospho-AKT (1:1000, Cell Signaling Technology), rabbit anti-phospho-STAT3 (1:1000, Cell Signaling Technology), mouse anti-total AKT (1:1000, Santa Cruz), rabbit anti-ADA1 (1:500, Abcam), mouse anti-beta-actin (1:1000, Santa Cruz) and mouse anti-α-Tubulin (TU-02) (1:400, Santa Cruz). Fluorescent anti-mouse/rabbit secondary antibodies were used (1:15000, LI-COR Biosciences). The bound antibodies were visualized and quantified with a LI-COR Odyssey Fc system (LI-Cor Biosciences, Lincoln, USA). Quantitative analysis of band densities was normalized to α-Tubulin unless otherwise stated. Data were expressed as fold change compared with control.

### Adenoviral-mediated ADA1/ADA2 overexpression

We used adenoviral vectors expressing the human ADA1 gene, combined with hrGFP in a polycistronic IRES construct (Vectorbuilder), and the human ADA2 gene (Flag-tagged, Vigene Biosciences), both behind CMV promoters. An adenoviral vector with only EGFP expression was used as control virus. The ADA1 adenoviral vector was titrated to generate low, intermediate and high hrGFP expression. Using 250 IFU/cell we obtained 35% hrGFP-positive cells with low GFP intensity, 1000 IFU/cell generated 53% hrGFP-positive cells with intermediate GFP-intensity, and 2000 IFU/cell resulted in 65% hrGFP-positive cells with high intensity 48h after transduction. The control virus particles were titrated to generate similar EGFP expression as the ADA1 vector (84% EGFP-positive cells), which was obtained at 10 IFU/cell. The commercial ADA2 adenoviral vector was not constructed to express bicistronic GFP, and we could not detect ADA2 using available antibodies (results not shown). Instead, this vector was titrated for phenotypic effects on cell viability avoiding decreases of basal cell viability, which occurred at concentrations not exceeding 25 VP/cell. For the adenoviral-mediated transduction, EndoC-betaH1 cells were incubated at 37°C for 4 h in 48 well culture plates in a volume of 0.25 ml culture medium. One day after transfection culture medium was changed to contain additives such as adenosine or palmitate + high glucose.

### Quantification of inosine and adenosine

For determination of intracellular nucleoside levels EndoC-betaH1 were cultured at control condition (cultured as described above) or with the addition of 1.5 mM sodium palmitate (2% fatty acid free albumin) + 22 mM glucose for 6 h or 24 h. After the treatment, cells were washed in ice-cold PBS, homogenized in iced-cold 0.4 M perchloric acid, and then centrifuged at 13,000 × g for 10 min at 4°C. Supernatants were collected and neutralized by adding 1:10 volume of 4 M potassium carbonate solution. Neutralized supernatants were centrifuged at 13,000 x g for 10 min at 4°C prior to analyze for intracellular adenosine level by using the human adenosine ELISA kit from MyBiosource (Catalog #: MBS2605344) and for inosine/hypoxanthine concentrations using the Inosine Assay Kit (Sigma-Aldrich, catalog # MAK100) according to manufacturer’s protocols. The human adenosine ELISA kit detects only adenosine, and not 2’-deoxyadenosine, and the Inosine Assay Kit detects both inosine and hypoxanthine.

For determination of extracellular inosine levels cells were, after the viral transduction period, incubated in a KRBH buffer containing the same glucose concentration as during the culture and 50 µM adenosine (substrate for the ADA reaction). Samples were taken from the KRBH buffer at 20 and 120 minutes and analysed using the inosine/hypoxanthine Assay Kit given above.

### siRNA treatment

ADA1 siRNA (12 nmol, ON-TARGETplus SMARTpool human ADA, Dharmacon) or a Mission negative control (scrambled) siRNA (Sigma) were used to transfect an estimated 50x10^3^ EndoC-betaH1 cells in 48 well plates. SiRNA was diluted in 25 μL of Opti-MEM and mixed with 0.5 μL of Lipofectamine™ RNAiMAX (Invitrogen, USA) diluted in 25 μL of Opti-MEM. The total mix was incubated for 10 min at room temperature and then diluted in a total of 300 μL of OptiMEM transfection medium to obtain a final siRNA concentration of 40 nM. The cells were incubated for 4 hours at 37°C. Transfection medium was replaced after 4h by normal culture medium.

### miR-30e-3p binding site inhibitor

An miRCURY LNA miRNA Power Target Site Blocker of miR-30e-3p binding to the ADA1 mRNA 3’-untranslated region (3’-UTR) (HS_ADA_30e-3p, YT0071418-EDA) and a scrambled negative control (Neg control, YT0070373-EDA) were obtained from QIAGEN (Hilden, Germany). EndoC-betaH1 cells were treated with 400 nM of the two oligonucleotides for 30 hours, using no lipofection, and then exposed to adenosine (0.5 mM) or high glucose + palmitate for another 18 hours, with the two oligonucleotides remaining in the culture medium. Cell death rates and intracellular adenosine levels were determined as given above.

### Data analysis

Results were presented as means ± SEM. Statistical analysis was done with GraphPad Prism Version 6.0c (GraphPad software, CA, USA). Statistical significance among several groups was analyzed, unless stated otherwise, by using repeated measurement one- or two-way ANOVA followed by the Sidak multiple comparison post-hoc test. P < 0.05 was considered statistically significant. *, **, *** and **** denote P < 0.05, P < 0.01, P < 0.001 and P < 0.0001, respectively.

## Results

### Adenosine and 2’-deoxyadenosine increase human beta-cell death

**Figure 1:**
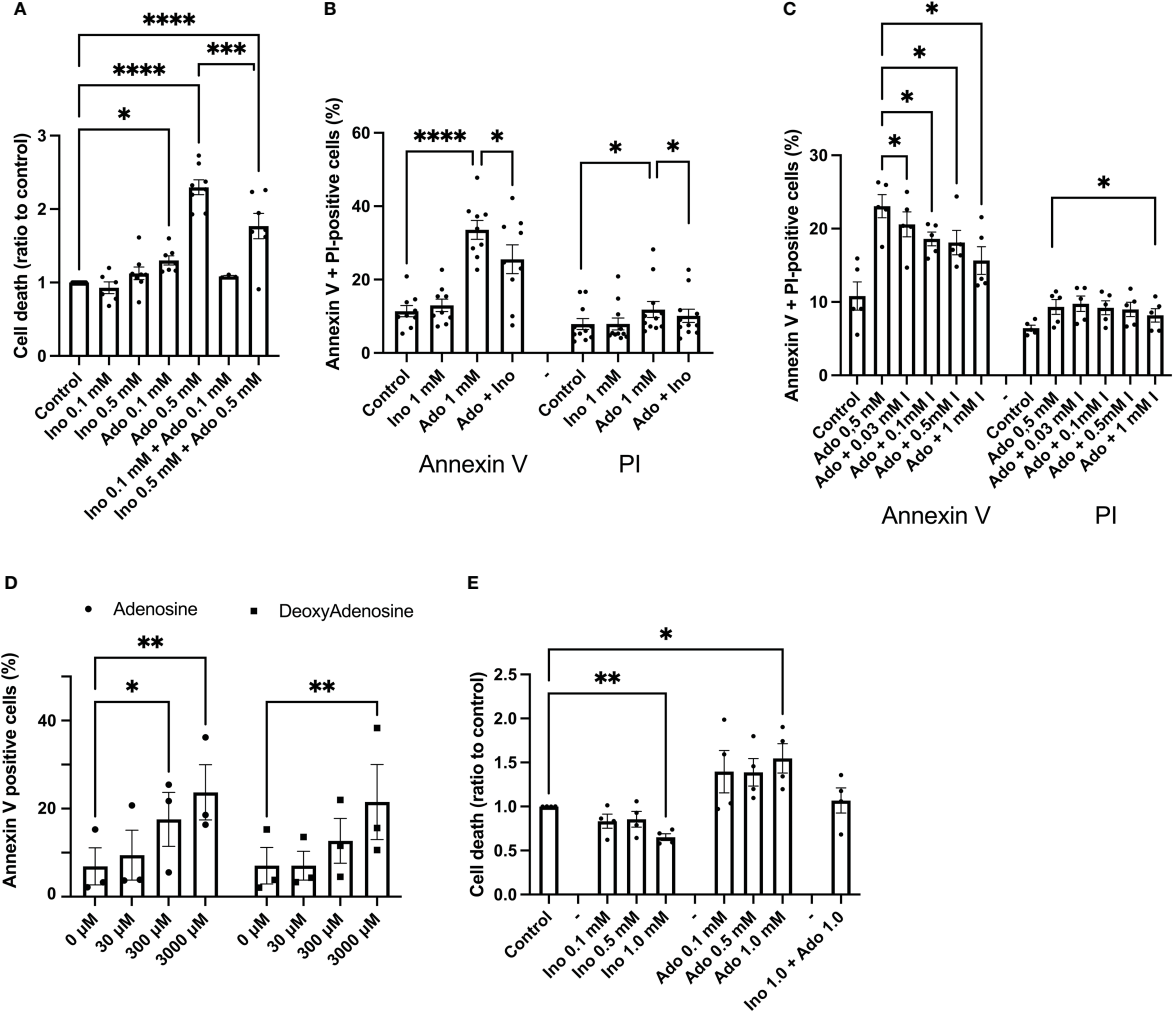
Adenosine, but not inosine, increases EndoC-betaH1 and human islet cell death. **(A)** EndoC-betaH1 cells were incubated for 48h with adenosine (Ado, 0.1 and 0.5 mM), inosine (0.1 and 0.5 mM) or adenosine + inosine. Cells were then stained with PI and analysed by flow cytometry. **(B)** EndoC-betaH1 cells were incubated for 24h with adenosine and inosine (1 mM), and stained with Annexin V + PI for flow cytometry analysis. **(C)** EndoC-betaH1 cells were incubated for 24h with 0.5 mM adenosine in the presence of increasing concentrations of inosine, and analyzed by Annexin V + PI staining and flow cytometry. **(D)** EndoC-betaH1 cells were incubated for 24h with increasing concentrations of adenosine and 2-deoxyadenosine, and analyzed with Annexin V + PI staining and flow cytometry. **(E)** Human islets were incubated for 48h with increasing concentrations of adenosine and inosine, and staining with PI (dead cells) +Hoechst (all cells) followed by fluorescence microscopic quantification of PI and Hoechst stain. The PI/Hoechst ratio was calculated as a relative measure of islet cell death.

EndoC-betaH1 cells were exposed to adenosine and inosine for 48h and then stained with propidium iodide (PI), a marker for damaged cell membranes. 100 μM adenosine increased EndoC-betaH1 cell death moderately, whereas 500 μM adenosine promoted a strong effect (**Figure 1A**). Inosine by itself did not affect cell death, but reduced adenosine-induced cell death when combined at equimolar concentrations (**Figure 1A**). To test whether adenosine kills cells by apoptosis or necrosis, we stained cells with both Annexin V, which identifies early and late apoptosis, and PI, which allows the identification of necrotic and late apoptotic cells. An 18h adenosine incubation of EndoC-betaH1 cells increased both Annexin V positive cells and PI positive cells (**Figure 1B**). The Annexin V increase, however, was bigger than the PI increase, suggesting that adenosine promotes mainly apoptosis and that some of the apoptotic cells also stain for PI when they become late apoptotic/necrotic. Again, inosine partially counteracted the apoptotic effect of adenosine (**Figure 1B**). We also tested different inosine concentration to see the dose-dependency of inosine-induced mitigation of the adenosine effect. Inosine protected already at 30 μM against apoptosis and the beneficial effect of inosine increased gradually with increasing dose up to 1 mM (**Figure 1C**). This suggests that inosine acts as a competitive adenosine antagonist.

We next compared the effects of adenosine with those of 2’-deoxyadenosine, which is a very weak adenosine receptor activator, as compared to adenosine. At 30 μM, a concentration of adenosine that activates adenosine receptors maximally, both adenosine and 2’-deoxyadenosine failed to affect beta-cell apoptosis (**Figure 1D**). However, at 3 mM, both compounds increased apoptosis similarly, suggesting that adenosine acts intracellularly rather than *via* extracellular receptors (**Figure 1D**). We also studied effects of a 24 h adenosine exposure on human islet cell death, using the PI staining method, and observed increased basal islet cell death in response to 1 mM adenosine, and decreased islet cell death in response to 1 mM inosine (**Figure 1E**). Interestingly, cell death was not increased in islets exposed to both adenosine and inosine. This demonstrates that adenosine induces beta-cell apoptosis at high concentrations that are above those that cause receptor-mediated effects.

### Adenosine and inosine are efficiently taken up by EndoC-betaH1 cells

To determine whether the uptake of extracellular adenosine and inosine differs in EndoC-betaH1 cells, we exposed cells at room temperature for 1 and 60 minutes to adenosine and inosine ^3^H isotopes in the presence of either 3 μM or 3 mM unlabeled adenosine/inosine. We observed that both adenosine and inosine were modestly taken up at 1 min and at a low extracellular concentration (3 μM), and that the uptake was increased approximately 60-fold after 60 min (**Suppl. Figure 1**). This supports the notion that there exists an active transport mechanism (CNT) for adenosine and inosine in beta-cells and that this transport mechanism has a rather low capacity. The uptake of 3 mM adenosine and inosine were dramatically higher at 1 min. This reveals that there also exists a passive transport mechanism *via* ENT1 and ENT2, and that these transporters are equally efficient for adenosine and inosine uptake. As adenosine and inosine are known to be taken up by the same transporters, ENT1 and ENT2 (2), it is likely that inosine competitively reduces adenosine uptake.

### Adenosine and inosine do not affect beta-cell extracellular acidification and oxygen consumption rates differently

It has been reported that adenosine can be metabolized and function as a nutrient (25). To test whether adenosine and inosine promote different metabolic effects on beta-cells, we studied oxygen consumption rates (OCR) and extracellular acidification rates (ECAR) in EndoC-betaH1 cells. A low concentration of adenosine and inosine (0.15 mM), injected acutely for 30 min, did not affect ECAR or OCR, either at basal conditions (results not shown), or at conditions of maximally stimulated OCR using the uncoupling compound FCCP (**Suppl. Figure 2**). This lack of effect was present both at low glucose (2.8 mM) and at high glucose (20 mM). A high concentration of adenosine or inosine (3 mM), however, both reduced maximal OCR in the presence of 2.8 mM glucose (**Suppl. Figure 2**). A similar trend was observed at 20 mM glucose. This suggests that adenosine does not promote beta-cell death *via* a different metabolism than that of inosine.

### Adenosine and 2’-deoxyadensine reduces plasma membrane PI [3,4,5]P3 levels

**Figure 2:**
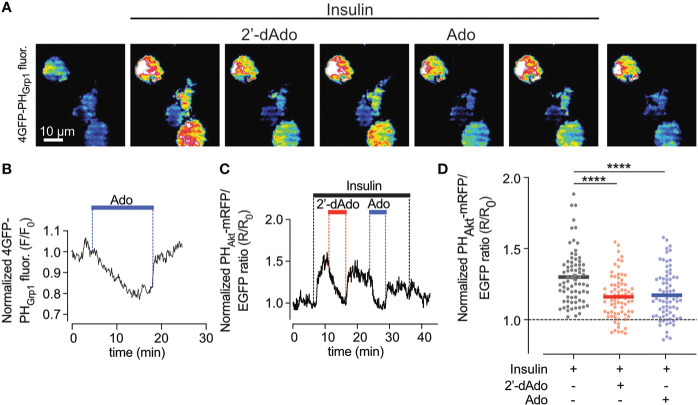
Adenosine and deoxyadenosine reduce plasma membrane PI(3,4,5)P_3_ levels. **(A)** TIRF microscopy images of four EndoC-β1 cells expressing GFP_4_-GRP1_PH_ following exposure to the indicated drugs. **(B)** TIRF microscopy recordings of GFP_4_-GRP1_PH_ fluorescence from a EndoC-betaH1 cell exposed to 1mM adenosine (Ado). **(C)** TIRF microscopy recording of PH_Akt_-mRFP/EGFP ratio in a EndoC-betaH1 cell sequentially exposed to 100 nM insulin, 1mM of deoxyadenosine (2’dAdo) and 1 mM adenosine (Ado). Note that the addition of insulin stimulated plasma membrane PI(3,4,5)P_3_ production, which was supressed by both adenosine and deoxyadenosine. **(D)** Quantification of the relative GFP_4_-GRP1_PH_/mCherry and PH_Akt_-mRFP/GFP ratio change at the plasma membrane of EndoC-betaH1 in response to 100 nM insulin or the combination of insulin and adenosine or deoxyadenosine. Quantifications of the GFP_4_-GRP1_PH/_mCherry and PH_Akt_-mRFP/EGFP from EndoC-betaH1 cells are presented as means ± S.E.M. for the indicated number of cells. (n=75 cells per group; ****P < 0.001; paired, 2-tailed Student’s t-test).

As the PI3-kinase-Akt pathway is a powerful controller of beta-cell apoptosis, we next studied effects of adenosine on plasma membrane PI(3,4)P_2_/PI(3,4,5)P_3_, which are the lipid products of PI3-kinase activity. To monitor changes in the plasma membrane concentration of PI(3,4)P_2_/PI(3,4,5)P_3_ in real time we used TIRF microscopy and the GFP-tagged PH_Grp1_ or mRFP-tagged PH_Akt_ domains (23), which translocate to the plasma membrane in response to insulin-induced activation of PI3-kinase with ensuing formation of the signalling lipids (**Figure 2A**). In unstimulated EndoC-betaH1 cells, the addition of adenosine (1 mM) caused an immediate reduction in plasma membrane GFP-PH_Grp1_ fluorescence, indicating a reduction in basal PI(3,4,5)P_3_ levels (**Figure 2B**). Next, we co-expressed a red PI(3,4)P_2_/PI(3,4,5)P_3_ sensor (mRFP-PH_Akt_) with cytosolic GFP as a reference marker in EndoC-betaH1 cells and exposed them to 100 nM insulin. This resulted in a marked increase in plasma membrane PI(3,4)P_2_/PI(3,4,5)P_3_ levels, seen as an increase in mRFP-PH_Akt_/GFP fluorescence ratio, and both adenosine and 2’-deoxyadenosine strongly reduced the insulin-induced increase in plasma membrane PI(3,4)P_2_/PI(3,4,5)P_3_  (**Figures 2B–D**). After wash-out of the purines PI(3,4)P_2_/PI(3,4,5)P_3_ levels returned to stimulated levels.

### Adenosine decreases Akt phosphorylation and down-stream signaling events

**Figure 3:**
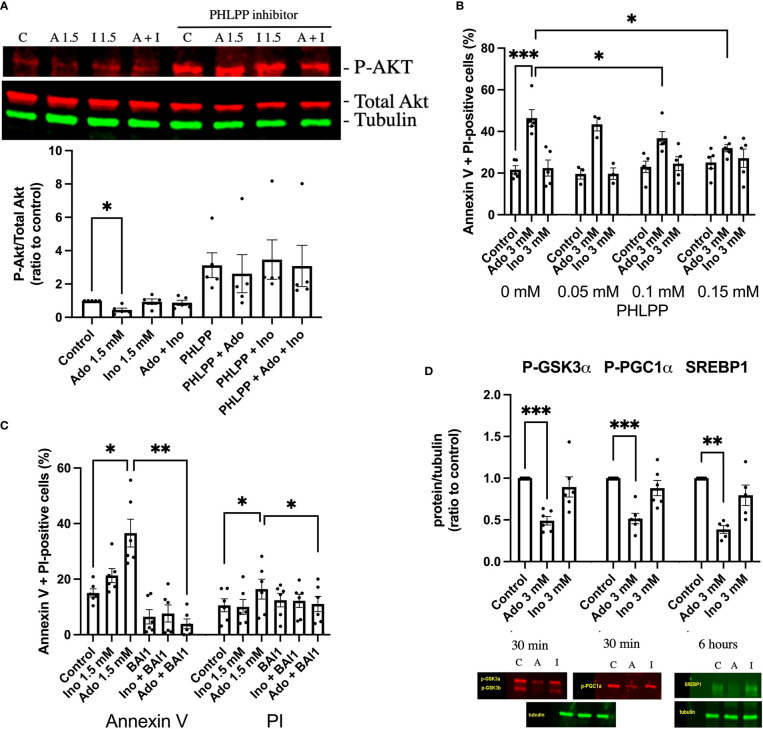
Adenosine-induced inhibition of Akt leads to increased beta-cell death. **(A)** EndoC-betaH1 cells were incubated for 30 min with 1.5 mM adenosine (A1.5), 1.5 mM inosine (I1.5) with and without 0.15 mM PHLPP inhibitor, and analyzed by immunoblotting for P-Akt. P-Akt signals were normalized to total Akt. **(B)** EndoC-betaH1 cells were incubated for 18h with 3 mM adenosine or inosine with increasing concentrations of PHLPP inhibitor, and stained with Annexin V and PI for flow cytometry analysis of total cell death rates. **(C)** EndoC-betaH1 cells were incubated for 18h with 1.5 mM of adenosine or inosine, with or without 5 μM of BAI1. **(D)** EndoC-betaH1 cells were incubated for 30 min or 6h with adenosine or inosine (3 mM) and analyzed by immunoblotting for P-GSK3α, P-PGC1α and SERBP1. Phospho-protein signals were normalized to tubulin.

Having observed decreased PI(3,4,5)P_3_ levels in response to adenosine and 2’-deoxyadenosine, we continued by analyzing phosphorylation of Akt at amino acid Ser308, an event that signals Akt activation and cell survival. We observed that a 30 min exposure to 1.5 mM adenosine, but not inosine, decreased Akt phosphorylation and that inosine partially counteracted this effect (**Figure 3A**). An inhibitor of the Akt dephosphorylating enzyme PHLPP (NSC117097) increased Akt phosphorylation dramatically in both control and adenosine treated cells (**Figure 3A**). As adenosine-induced inhibition on Akt phosphorylation could be reverted by the PHLPP phosphatase inhibitor, we next studied the effects of the inhibitor on cell survival. We observed that the phosphatase inhibitor dose-dependently inhibited adenosine-induced EndoC-betaH1 cell apoptosis + necrosis (**Figure 3B**). Thus, adenosine-induced apoptosis occurs *via* inhibition of the Akt pathway. We next treated cells with the Bax inhibitor BAI1 and found that adenosine-induced apoptosis was blocked by this inhibitor (**Figure 3C**). This indicates that adenosine-induced apoptosis occurs *via* the intrinsic apoptotic pathway. To further substantiate that adenosine downregulates Akt signaling we studied the phosphorylation of the Akt target GSK3 at position Ser9/21. We observed that this phosphorylation event was inhibited by adenosine after a 30 min exposure (**Figure 3D**), but not by inosine. Loss of Ser9/21-GSK3 phosphorylation results in enzyme activation and increased apoptosis (26). We also investigated the phosphorylation of PGC1α (Ser570), a transcription factor that controls mitochondrial biogenesis, and which is directly phosphorylated by Akt (27). This phosphorylation event was also inhibited by adenosine (**Figure 3D**). However, a 30 min exposure to adenosine did not affect the phosphorylation of AMPK (Thr172), eIF2α (Ser51) and STAT3 (Ser727) (**Suppl. Figure 3**), suggesting that adenosine targets the PI3K pathway selectively, without promoting a global suppressive effect. Additionally, after 6h of adenosine exposure we determined levels of the SREBP1 protein, a transcription factor that controls lipid biogenesis (28). SREBP1 was reduced by adenosine (**Figure 3D**), but not by inosine. SREBP1 expression is controlled by Akt (28), supporting the notion that adenosine attenuates this pathway as well. Taken together, these findings suggest that adenosine affects important signaling events that control beta-cell death, glycogen synthesis, lipid biosynthesis and mitochondrial biogenesis, all by inhibition of the PI3K-Akt signaling pathway.

### Palmitate + high glucose increases adenosine and inosine in EndoC-betaH1 cells

**Figure 4:**
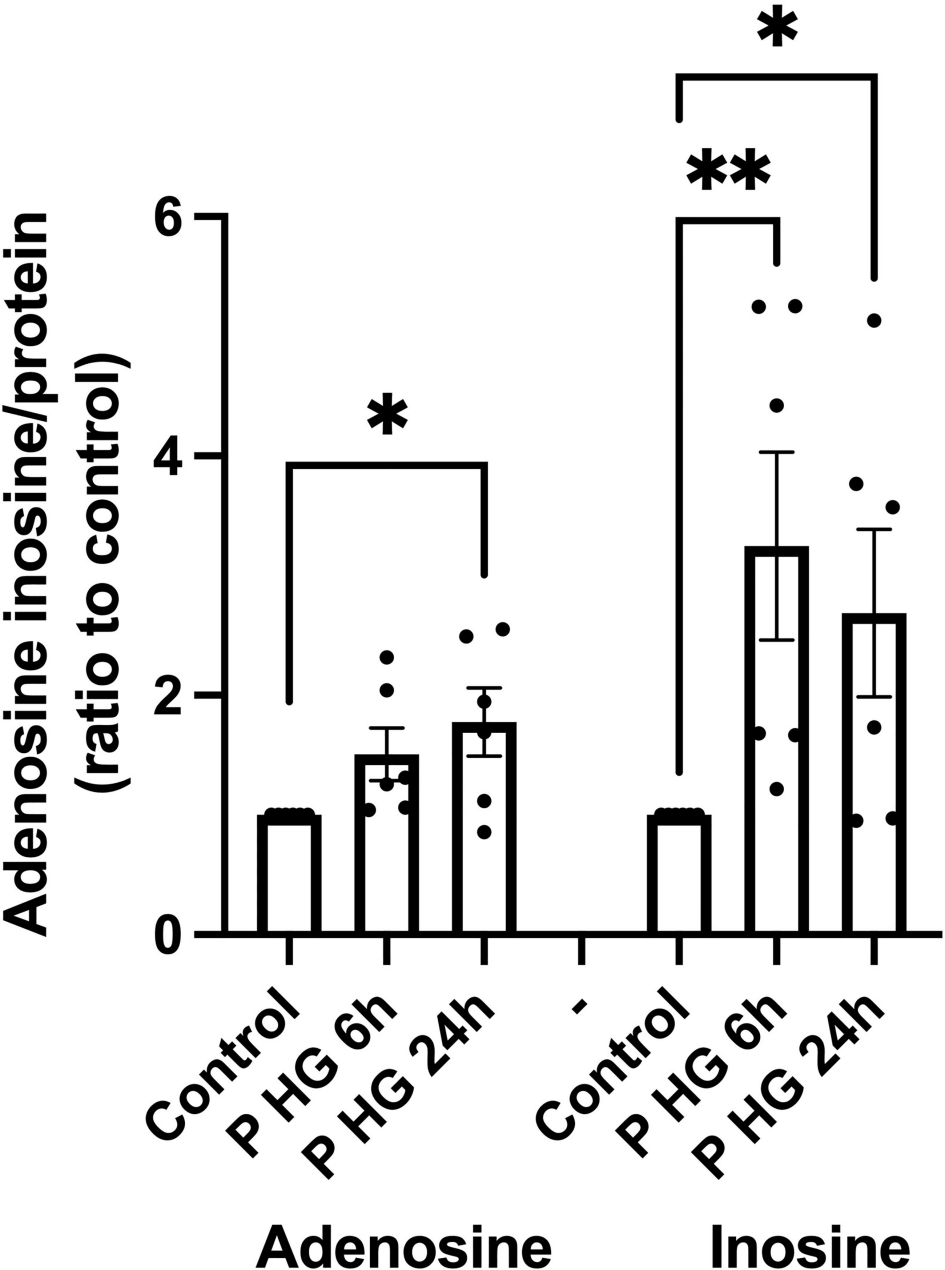
Palmitate + high glucose increases adenosine and inosine levels' **(A)** *Intracellu*lar adenosine and inosine were quantified in EndoC-betaH1 cells after incubation for 6 or 24h with palmitate (1.5 mM solubilized in 2% BSA) + high glucose (20 mM) (P HG). Adenosine was quantified using an adenosine ELISA and inosine was quantified using an Inosine fluorometric assay kit. Results were normalized to protein contents of the samples.

Palmitate + high glucose (P + HG), which together promote beta-cell metabolic stress and death, have been reported to reduce ATP in beta-cells (29), and could therefore cause an increase in intracellular adenosine levels. The cells were incubated for 6 or 24h with P + HG after which the intracellular contents of adenosine and inosine were determined. P + HG promoted a modest increase in intracellular adenosine at 24h and a more pronounced increase in inosine at both 6 and 24h (**Figure 4**). This suggests that adenosine is increased, probably due to ATP/ADP/AMP dephosphorylation, and that some of the adenosine/AMP is deaminated to inosine/IMP *via* ADA/AMPD-catalyzed deamination. The effects of metabolic stress on 2’-deoxyadenosine levels are unknown as the adenosine ELISA kit does not detect 2’-deoxyadenosine.

To test whether increased intracellular inosine modulates beta-cell death rates, we next incubated EndoC-betaH1 cells for 18h with palmitate + high glucose with or without increasing concentrations of inosine. We observed that inosine promoted only a small protective effect against beta-cell necrosis at control conditions, but that apoptosis and total cell death were not affected by inosine, neither at control conditions nor with palmitate + high glucose (**Suppl. Figure 4**). Thus, increased levels of inosine are of little importance for beta-cell survival in other situations than when adenosine is high extracellularly so that extracellular inosine competes with adenosine for uptake into the cells, thereby ameliorating the deleterious effects of adenosine.

### Overexpression of ADA1, but not of ADA2, protects EndoC-betaH1 cells against both adenosine and palmitate + high glucose-induced apoptosis

**Figure 5:**
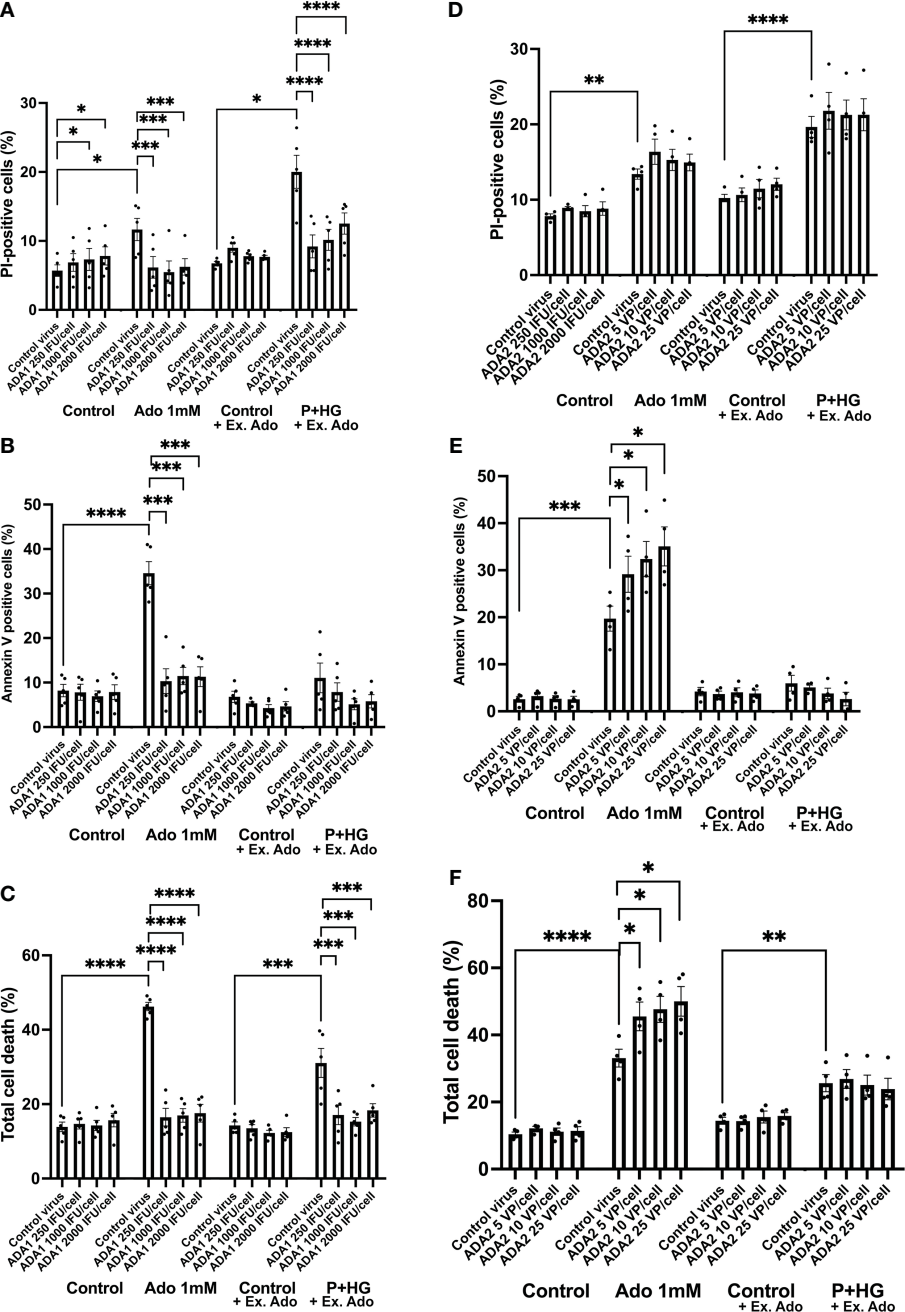
Overexpression of ADA1, but not ADA2, protects against adenosine and palmitate + high glucose-induced EndoC-betaH1 cell death. EndoC-betaH1 cells were transduced with ADA1 **(A–C)** and ADA2 **(D–F)** adenoviral vectors at the concentrations given in the Figure. The next day, cells were incubated at control conditions or with adenosine (Ado 1 mM) or palmitate + high glucose (P+HG, 1.5 mM palmitate + 20 mM glucose) for an additional 18h. Controls to the P+HG groups and the P+HG groups were supplemented with 50 μM adenosine. Cell apoptosis (Panels and necrosis rates were then analyzed by PI **(A, D)** and Annexin V staining **(B–E)** and flow cytometry. Total cell death **(C, F)** was calculated by adding together PI and Annexin V cell percentages.

To further support the notion that intracellular adenosine promotes beta-cell death in response to metabolic stress, we overexpressed, by adenoviral transduction, the two adenosine deaminase enzymes ADA1 and ADA2 and studied the effect on adenosine- and palmitate + high glucose-induced beta-cell death. An intracellular surplus of adenosine is cleared from cells either by release to the exterior, or by deamination to inosine, an irreversible reaction catalyzed by the ADA enzymes, which leads to the sequential formation of hypoxanthine, xanthine and finally uric acid. ADA1 transduction resulted in ADA1 expression as assessed by immunoblot analysis (**Suppl. Figure 5A**). A high adenosine concentration (1 mM) evoked a small increase in PI-positive cells (necrosis, **Figure 5A**) and a large increase in annexin V positive cells (apoptosis, **Figure 5B**), whereas P + HG increased predominantly necrosis (**Figure 3A**). ADA1 overexpression promoted a weak increase in PI-positive cells at control conditions (**Figure 5A**, control to adenosine groups). To explain this, we hypothesized that strong ADA1 overexpression leads to depletion of cellular adenosine, AMP, ADP and ATP, and that this leads to an energy crisis and necrotic cell death. We therefore supplemented the control cells to the palmitate + high glucose groups with a non-toxic dose of extracellular adenosine (50 μM), and observed that when there is a sufficient supply of extracellular adenosine EndoC-betaH1 cells do not die in response to ADA1 overexpression (**Figure 5A**). As we sought to investigate the effects of excess adenosine, and not adenosine deficiency, both control and P + HG exposed cells were supplemented with 50 μM adenosine. ADA1 overexpression, at all viral vector doses, counteracted completely both high adenosine- and P + HG-induced apoptosis and necrosis (**Figures 5A, B**) resulting in protection against total cell death in both cases (**Figure 5C**). This powerful effect of ADA1 strongly suggests that ADA1 efficiently converts adenosine to the more inert nucleoside inosine, and that intracellular adenosine participates in palmitate + high glucose induced cell death. Interestingly, ADA1 overexpression resulted in increased Akt phosphorylation, both at basal conditions and in adenosine exposed EndoC-betaH1 cells (**Suppl. Figure 6**), suggesting that ADA1-induced adenosine deamination results in increased Akt signaling. Transduction of EndoC-betaH1 cells with ADA2, however, did not affect basal death rates, nor was there any protection against 1 mM adenosine (**Figures 5D–F**). Instead, ADA2 overexpression potentiated adenosine-induced apoptosis and total cell death rates (**Figures 5E, F**). Furthermore, ADA2 did not protect against palmitate + high glucose induced necrosis (**Figure 5D**).

### Overexpression of ADA1, but not of ADA2, increases inosine/hypoxanthine levels in EndoC-betaH1 cells

**Figure 6:**
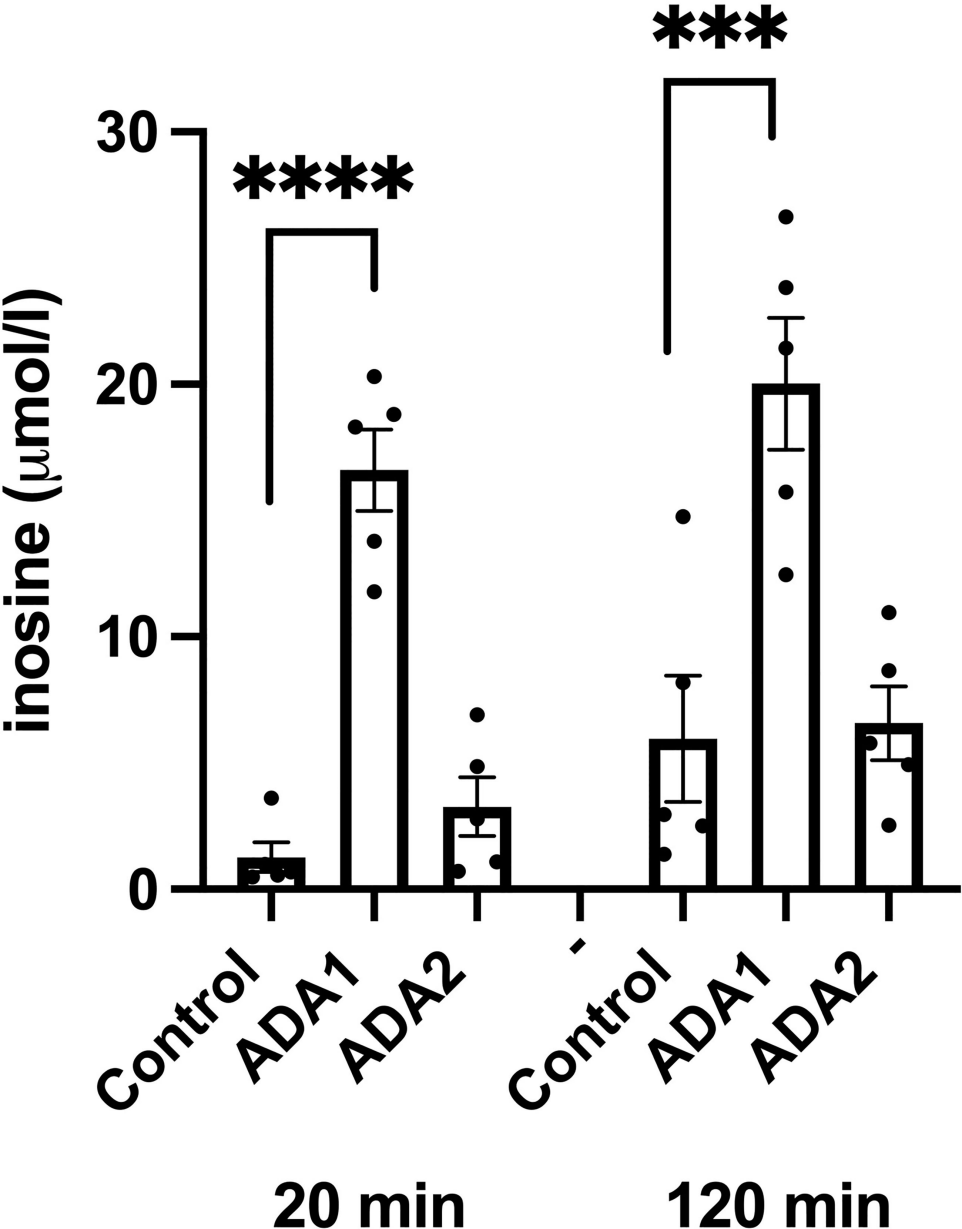
ADA1 overexpression, but not ADA2 overexpression, leads to increased inosine production. EndoC-betaH1 cells were transduced with ADA1 (2000 IFU/cell) or ADA2 (25 VP/cell) and analyzed for inosine production the next day in the presence of 50 μM adenosine. Results are inosine concentrations in the extracellular KRBH buffer.

We next studied effects of ADA1 (1000 VP/cell) and ADA2 (25 IFU/cell) overexpression on inosine/hypoxanthine levels. EndoC-betaH1 cells, transduced with ADA1 or ADA2 adenoviral particles, were pre-cultured for 18 hours and then incubated in the presence of 50 μM adenosine for 20 and 120 minutes. As we observed in separate experiments that the majority of inosine/hypoxanthine produced by EndoC-betaH1 cells during a 2h incubation period is transported extracellularly (results not shown), we determined in this case only extracellular inosine/hypoxanthine levels. ADA1 overexpression resulted in a dramatic increase in extracellular inosine/hypoxanthine reaching levels corresponding to the level of extracellular adenosine (**Figure 6**). This increase was present already after 20 minutes suggesting that ADA1 rapidly deaminates adenosine present in the incubation buffer. At 120 minutes, the inosine levels were not further increased by ADA1 overexpression, probably because most of the substrate for the deamination reaction had already been consumed during the first 20 min. ADA2 overexpression did not significantly affect inosine/hypoxanthine levels, either at 20 or at 120 min (**Figure 6**). These findings suggest that adenovirally transduced ADA2 does not efficiently convert adenosine to inosine in EndoC-betaH1 cells and that ADA1-associated protection against cell death correlates with increased adenosine deamination.

### siRNA-mediated downregulation of ADA1 increases cell death in EndoC-betaH1 cells

**Figure 7:**
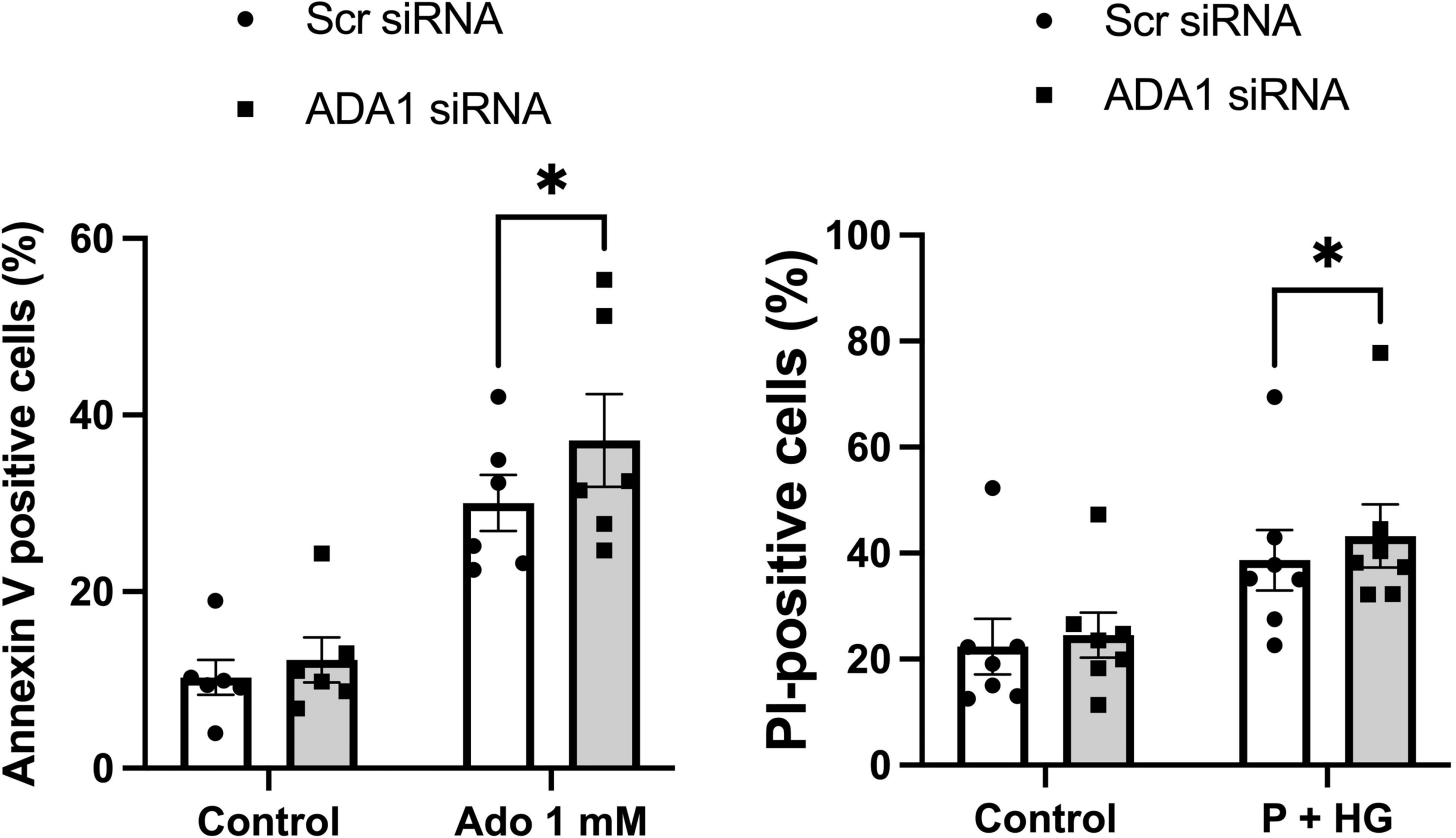
Knockdown of ADA1 results in increased cell death in response to adenosine or high glucose + palmitate. EndoC-betaH1 cells were lipofected with scramble (Scr) or ADA1 siRNA. Two days later cells were treated with 1 mM adenosine (Ado 1 mM) (left panel) or with palmitate + high glucose (P + HG) (right panel). The next day cells were analysed by flow cytometry.

Having observed that adenoviral-mediated deamination of adenosine to inosine leads to complete protection against metabolic stress (P+HG)-induced cell death, we next studied whether downregulation of ADA1 expression, which is anticipated to generate increased intracellular adenosine levels, results in enhanced cell death rates. Basal expression of ADA1 is weak using commercial antibodies, but a weak 40 kDa band could be observed after optimization of the immunoblotting procedure (**Suppl. Figure 5B**). This band was reduced by approximately 50% in cells treated with ADA1 siRNA (**Suppl. Figure 5B**). Furthermore, siRNA treatment against ADA1 resulted in increased apoptosis of EndoC-betaH1 cells cultured in the presence of 1 mM adenosine for 18 h (**Figure 7**). Also the death of cells exposed to high glucose + palmitate was increased in ADA1 siRNA treated cells (**Figure 7**). This suggests that endogenous ADA1 expression counteracts beta-cell death in response to metabolic stress and increased intracellular levels of adenosine.

### Inhibition of miR-30e-3p binding to the ADA1 mRNA UTR decreases intracellular adenosine and cell death rates

**Figure 8:**
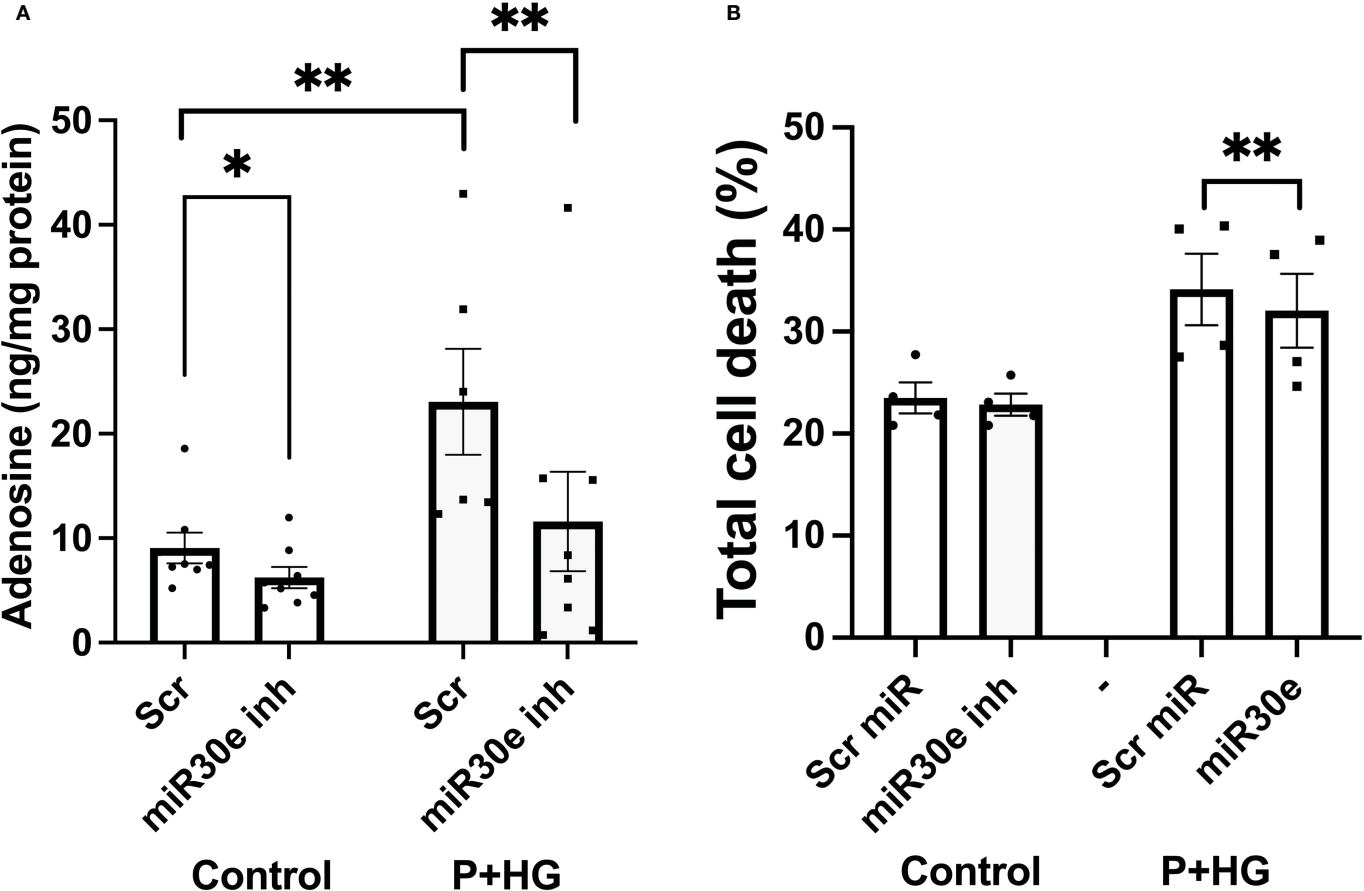
Inhibition of miR-30e-3p binding to the ADA1 3’-UTR leads to decreased intracellular levels of adenosine and improved cell survival EndoC-betaH1 cells were pre-treated for 30 hours with 400 nM of the miR-30e-3p target site inhibitor and then exposed to palmitate + high glucose (P+HG) over night **(A)**. Intracellular adenosine levels were analysed using an ELISA and expressed per mg protein. **(B)** Cells were treated as in **(A)** and then analysed by flow cytometry for quantification of Annexin V and PI positivity. Results are total cell death rates, i.e. the sum of the percentages Annexin V and PI positive cells.

Prediction of miRNA binding to the ADA1 mRNA 3’-UTR, using the mirdb.org site, revealed that 14 different miRNAs could bind this target (**Suppl. Figure 7**). The two miRNAs with the highest target score, hsa-mir-4533 and hsa-mir-3663-3p, are not highly expressed in different human tissues (https://dianalab.e-ce.uth.gr/mited/#/expressions), and were therefore excluded from further studies. Instead, we chose to study hsa-mir-30e-3p, a member of the miRNA-30 family, which is highly expressed in beta-cells, according to the same database. Interestingly, members of the miRNA-30 family have been reported to affect beta-cell insulin production, apoptosis and transdifferentiation to alpha-cells, and to function as biomarkers for pre-diabetes (30–32). We observed that blocking the binding of hsa-mir-30e-3p to ADA1 mRNA resulted in reduced intracellular adenosine levels, both at control conditions and in cells exposed to palmitate + high glucose (**Figure 8A**). This was paralleled by a small but consistently improved cell survival at palmitate + high glucose conditions (**Figure 8B**). These findings suggest that beta-cell adenosine disposal is limited by miR-30e-3p, which results in a reduced beta-cell survival in response to metabolic stress.

## Discussion

**Figure 9:**
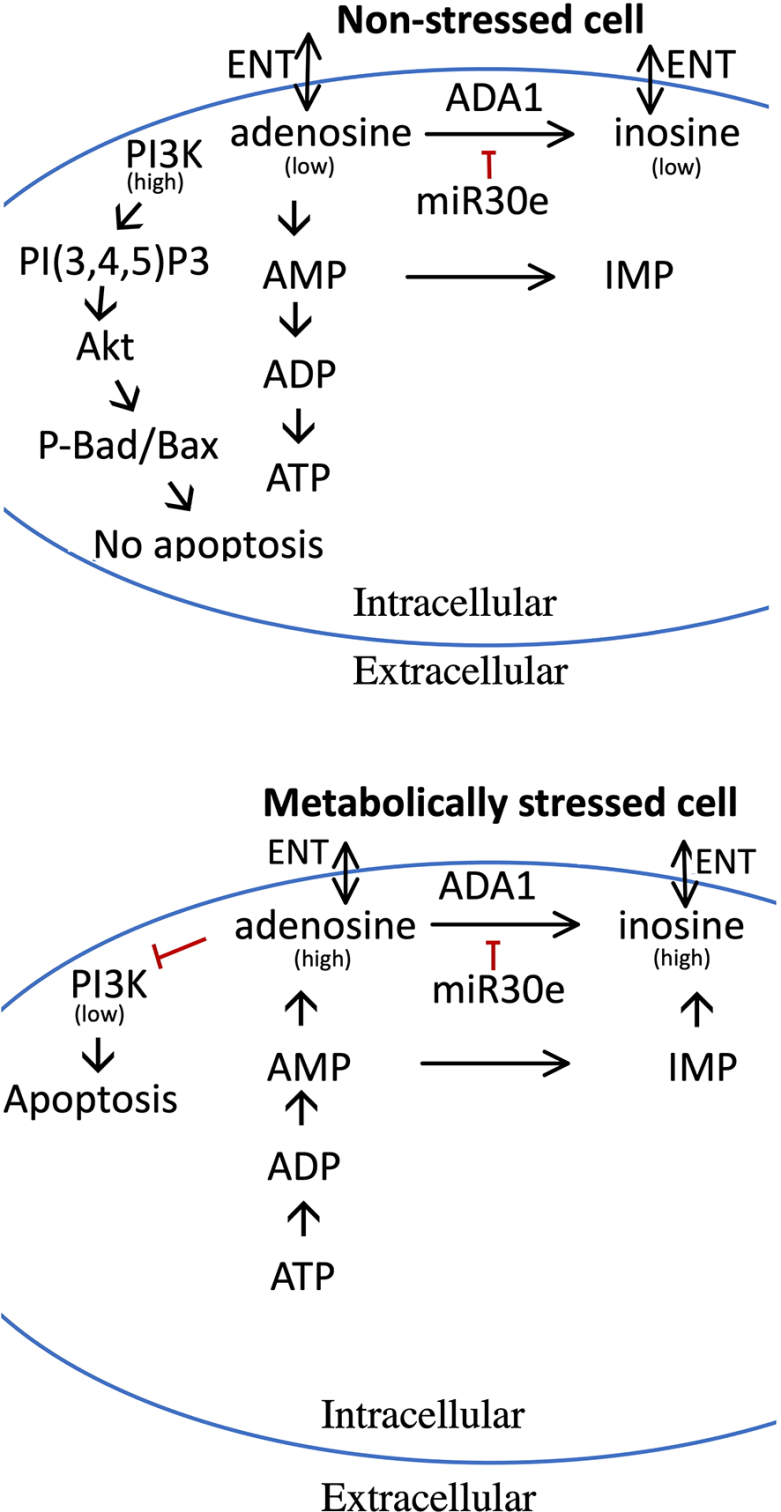
Model for the pro-apoptotic role of adenosine and how increased adenosine is metabolized in non-stressed and metabolically stressed beta-cells. Upper panel depicts adenosine metabolism in non-stressed cells, and the lower panel adenosine metabolism and pro-apoptotic effects in stressed cells. In stressed cells, nucleotide dephosphorylation leads to increased adenosine levels, which reduces the activity of the PI3-kinase. In non-stressed cells, adenosine is used for the maintenance of high levels of ADP and ATP, and adenosine levels therefore remain low with little or no effect on the PI3-kinase. High levels of adenosine can be reduced by ADA1 activity, leading to the production of inosine, or by AMP deamination to IMP. ADA1 activity is, however, reduced by miR-30e-3p. Both adenosine and inosine can be release to the exterior of the cell via the ENT transporters. See text for details.

In the present study we observe that high concentrations of adenosine or 2’-deoxyadenosine rapidly inhibits PI3K-mediated production of PI(3,4,5)P_3_ leading to lowered phosphorylation of Akt and GSK3, which in turn causes Bax-mediated apoptosis. The PI3K protein is known to contain an adenine-binding site, situated in the ATP pocket (33), and it is therefore easily envisaged that adenosine, by binding to this site, competitively prevents ATP from reaching the pocket and therefore also PI3K enzymatic activity. As intracellular adenosine was increased by palmitate + high glucose we propose that in non-stressed cells, in which adenosine is used mainly for synthesis of ATP (8), intracellular adenosine contents are low, which leads to that there are minimal effects on the anti-apoptotic PI3K-pathway. On the other hand, in metabolically stressed beta-cells adenosine levels are increased, *via* adenosine nucleotide dephosphorylation, causing to inhibition of the PI3K pathway and increased Bax-mediated apoptosis (**Figure 9**). The presently observed increase in intracellular adenosine of metabolically stressed cells was rather small. However, it is likely that severe metabolic stress occurs late in the palmitate + high glucose-induced cell death process, and that only a small fraction of the total cell population at a specific time point are overwhelmed with intracellular adenosine.

The assumption that it is intracellular, but not extracellular, adenosine which mediates these effects is based upon the observations that 2’-deoxyadenosine, which has been reported to lack agonistic effects on adenosine receptors (34), promoted similar effects as adenosine, and that apoptosis was seen at concentrations above those that activate adenosine receptors maximally. Furthermore, the protective effect of ADA1 occurred probably *via* disposal of intracellular adenosine, and not extracellular.

We identified ADA1 as an adenosine metabolizing enzyme that promotes strong anti-apoptotic effects. Indeed, efficient deamination of adenosine to inosine, by adenoviral ADA1 overexpression, rescued beta-cells from apoptosis at control conditions, from apoptosis in the presence of a high adenosine concentration, and from necrosis at conditions of metabolic stress (palmitate + high glucose). That necrosis dominates in metabolically stressed beta-cells and apoptosis in adenosine-exposed cells is explained by a severe lack of ATP in the former case, which limits the energy supply necessary for execution of the apoptotic program. Thus, activation of the intrinsic apoptotic pathway is probably initiated in both cases, but only cells with ATP can go through with the entire apoptotic program.

Expression of the ADA1 protein is low in beta-cells (undetectable by immunoblot analysis), but siRNA-mediated knockdown of ADA1 resulted in increased cell death rates promoted by adenosine or metabolic stress, suggesting that there exists a low basal expression of ADA1 and that this participates in the disposal of excess and toxic adenosine. Thus, ADA1 activity could therefore represent a critical and adjustable step in the adenosine metabolism of insulin producing cells and therefore also beta-cell survival. This resembles the situation in ADA-SCID (12), in which a genetic defect in the ADA1 gene results in increased levels of adenosine and 2’-deoxyadenosine, an early loss of T-, NK- and B-cells and severe immunodeficiency. That ADA1 may play a role also in the pathogenesis of T1D, and not only in ADA-SCID, is supported by the finding that polymorphisms in the ADA1 gene are associated with an increased risk for T1D (35, 36). Furthermore, in one case report it was described that a patient with mild ADA-SCID also developed diabetes (37). Interestingly, the patient lacked the typical autoantibodies and genetic markers that are usually seen in T1D, suggesting a non-autoimmune-mediated loss of beta-cells. Although it is likely that mild ADA-SCID promotes a dysregulated immune system and therefore an increased risk for an autoimmune attack on beta-cells and subsequent T1D (3), the present findings support the notion that a decreased adenosine disposal rate, caused for example by a lowered ADA1 activity, initiates non-autoimmune apoptosis/necrosis also in beta-cells, and not only in lymphocytes, and that this could contribute to the pathogenesis of T1D.

Due to technical limitations, we have not been able to analyze levels of 2’-deoxyadenosine in beta-cells. However, as it is likely that 2’-deoxyadenosine is increased during metabolic stress, and as 2’-deoxyadenosine inhibited PI3K activity equally well as adenosine, both 2’-deoxyadenosine and adenosine should probably be considered proapoptotic to beta-cells. Moreover, as 2’-deoxyadenosine is equally well deaminated as adenosine by ADA1 (12), it can be assumed that ADA1 rescues beta-cells by increasing disposal of both nucleosides. Further studies will hopefully improve our understanding of the relative importance of adenosine and 2’-deoxyadenosine in beta-cell death. Inosine, the deamination product of adenosine, however, seems to be rather inert when it comes to beta-cell survival, and its powerful antagonistic effect on adenosine-induced cell death is most likely due to competitive inhibition of adenosine uptake. This notion is supported by our finding that inosine did not protect against cell death in the absence of exogenous adenosine.

Overexpression of ADA2, however, failed to protect beta-cells, which fits well with previous characterization of ADA2 as an extracellular adenosine deaminase with a Km of approximately 2 mM (38). Therefore, it is likely that ADA2 plays a very insignificant role, if any at all, in the metabolism of adenosine in beta-cells. Instead, it has recently been reported that ADA2 functions as a growth factor (39) or possibly as an interferon-I response factor (40), which may explain why ADA2 overexpression instead potentiates beta-cell death in the presence of a high adenosine concentration.

The low expression level of the ADA1 protein in EndoC-betaH1 cells is probably explained, at least in part, by miRNA-mediated suppression of ADA1 translation. Indeed, using a miR-30e target blocker specific for the 3’UTR of ADA1 mRNA, we observed lowered intracellular adenosine levels and higher cell survival rates. It appears that the miRNA-30 family is particularly abundant in islet cells (41). Thus, due to the high islet miR-30e expression level, it may be that the beta-cell capacity to handle an adenosine buildup may be unnecessarily and unphysiologically restricted in situations leading to metabolic stress-induced apoptosis. It is not clear why the ADA1 level and activity appear to be attenuated by high levels of miRNA-30e, but it may be speculated that highly differentiated beta-cells need to maximize buildup of ATP and ADP, in order to efficiently regulate opening and closing of the ATP-sensitive potassium channel, and a continuous deamination of adenosine to inosine might hamper this insulin secretory process. It should be noted, however, that blocking the miR-30e target site mediated only a small protection against cell death *in vitro*. Thus, the significance of this mechanisms needs to be verified in follow-up studies.

In addition to ADA1, beta-cells can most likely utilize other enzymes/transporters, *i. e.* AMPD, which catalyzes the deamination of AMP to IMP, and ENT1/ENT2/ENBT1, which transport nucleosides over the cell membrane (**Figure 9**), for the disposal of adenosine (42–45), thereby alleviating the toxic effects of high levels of intracellular adenosine/2’-deoxyadenosine. It is likely that the activities of these enzymes/transporters, depending on the particular situation, are regulated by transcriptional/post-transcriptional/post-translational mechanisms (43, 44), and that factors or circumstances that cause suboptimal AMPD- and ENT-mediated adenosine disposal could be detrimental to beta-cells. Unfortunately, our knowledge on these complex events is far from satisfactory, and further studies are highly warranted to better understand the control of adenosine metabolism/efflux in beta-cells.

As it can easily be envisaged that beta-cell dysfunction and death in diabetes involves increased intracellular levels of adenosine and/or 2’-deoxyadenosine, it is tempting to propose that a pharmacological strategy to reduce beta-cell adenosine, for example *via* increased ADA1 activity, could lead to improved beta-cell function and survival in both T1D and T2D. Patients with ADA-SCID have in some cases been treated with long-circulating PEG-ADA, a replacement therapy consisting of the bovine ADA1 protein linked to polyethylene glycol *via* lysine bonds (3). Unfortunately, PEG-ADA reaches only the plasma compartment, and not the interior of cells, and treatment with PEG-ADA is known to promote unwanted side-effects and long-term complication (3). Thus, PEG-ADA may not be suitable for prevention or treatment of beta-cell dysfunction in diabetes, and other and more beta-cell specific strategies for improved adenosine disposal, for example by using miRNA target blockers, might be more attractive alternatives.

## Data availability statement

The raw data supporting the conclusions of this article will be made available by the authors, without undue reservation.

## Author contributions

All authors participated in generating and analysing data and revising or drafting the article. All authors have approved the final version to be published. AN, JC and NW are the guarantors of this work and, as such, had full access to all the data in the study and take responsibility for the integrity of the data and the accuracy of the data analysis.
