# Supplementary material for: Metabolic stress-induced human beta-cell death is mediated by increased intracellular levels of adenosine
Source: Front Endocrinol (Lausanne). 2023 Jan 25;14:1060675. doi: 10.3389/fendo.2023.1060675 (PMC9905624; doi:10.3389/fendo.2023.1060675)
Supplement: Supplementary file 1 [file DataSheet_1.pdf]

Supplemental Figures

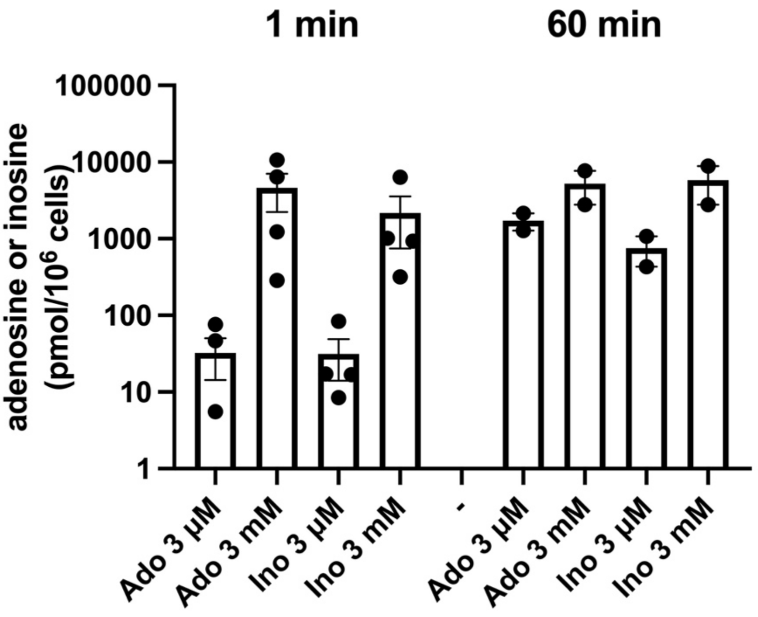

Suppl Fig 1

Uptake of ^{3}H-inosine is similar to that of ^{3}H-adenosine in EndoC-betaH1 cells.

EndoC-betaH1 cells were incubated for 1 or 60 min in the presence of tritiated adenosine or inosine at the concentrations 3 µM or 3 mM. Uptake of the nucleoside isotopes was quantified by scintillation counting. Results are means of 2-4 independent experiments.

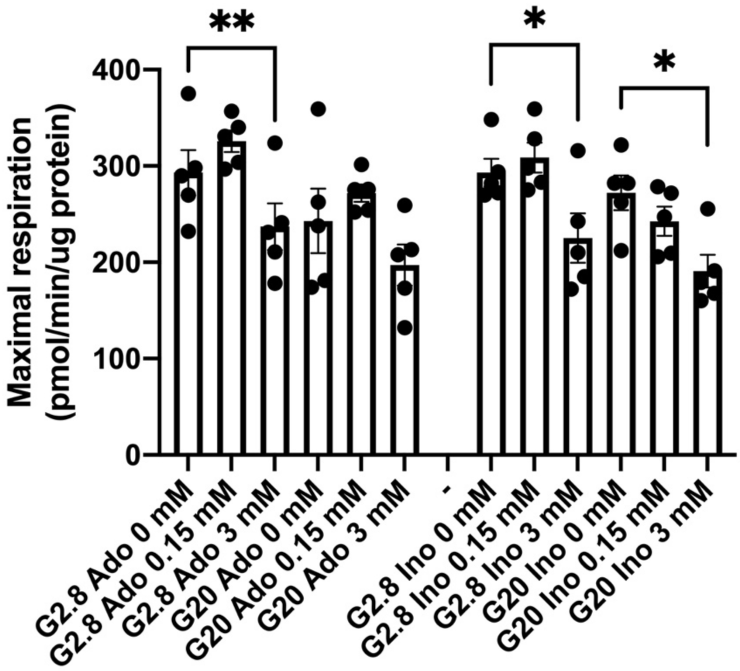

Suppl Figure 2

High concentrations of adenosine and inosine similarly reduce EndoC-betaH1 cell maximal respiration

EndoC-betaH1 cells were cultured for 48 h and then pre-incubated at 2.8 or 20 mmol/l glucose for 1 h prior to measuring mitochondrial respiration. Basal respiration was then measured with or without adenosine (Ado) or inosine (Ino) at 2.8 (G2.8) or 20 (G20) mM glucose. The adenosine and inosine were injected after 20 min of basal respiration recordings. Basal OCR (pmol/min, not shown), maximal OCR (after FCCP addition; pmol/min) and ECAR (mpH/min, not shown) were calculated. The results from five independent experiments are shown as means ± SEM.

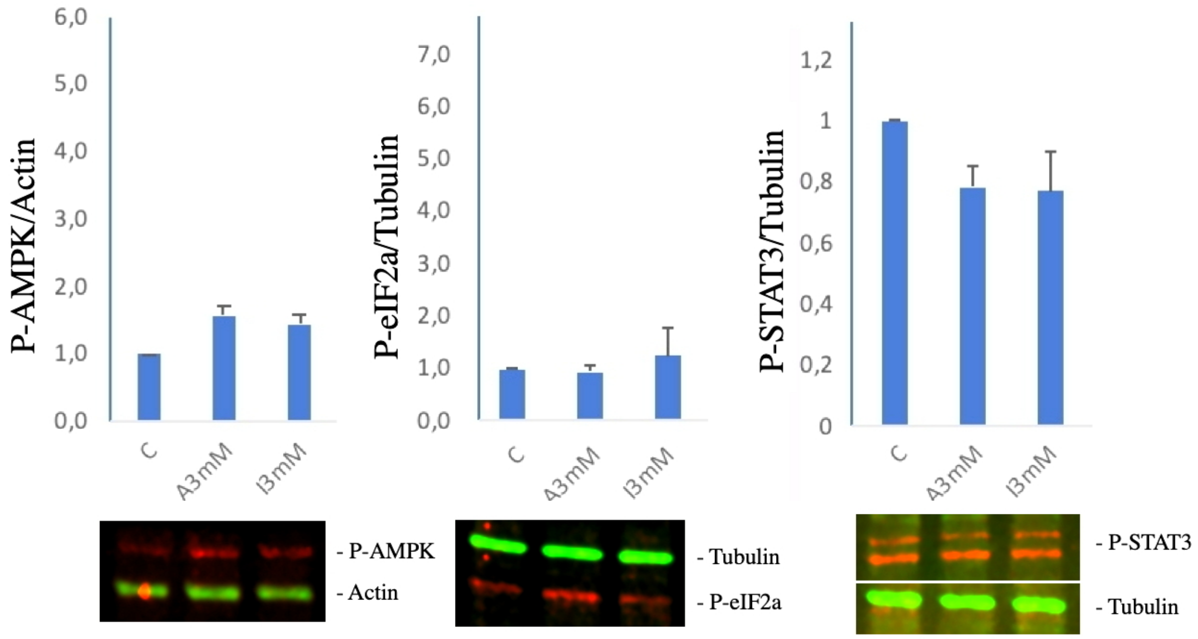

Suppl. Figure 3.

Adenosine does not affect EndoC-betaH1 P-AMPK, P-eIF2alpha and P-STAT3 levels

EndoC-betaH1 cells were incubated for 30 min with 3 mM adenosine (A3mM) or inosine (I3mM) and analyzed by immunoblotting for P-AMPK, P-eIF2alpha and P-STAT3. Results are from 3 separate experiments.

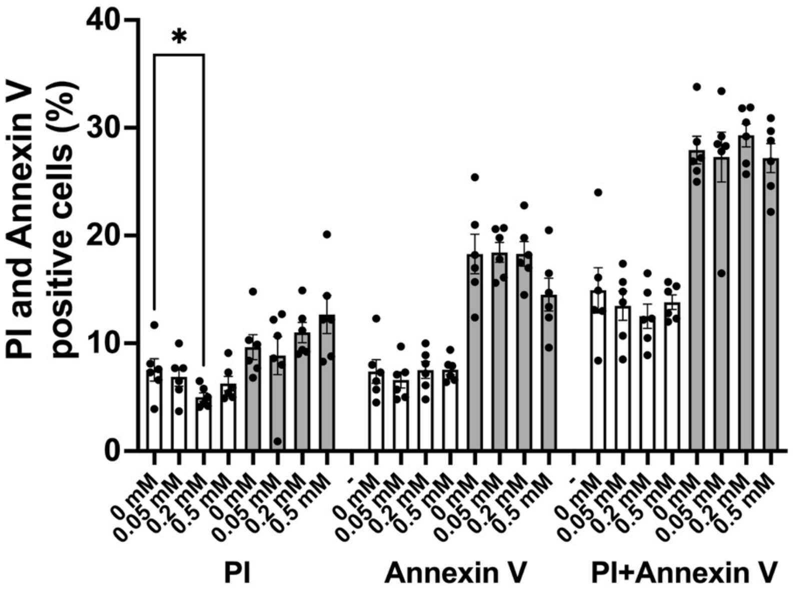

Suppl. Figure 4

Inosine does not protect against palmitate + high glucose-induced cell death.

Effects of increasing concentrations of inosine on EndoC-betaH1 cell PI and Annexin V staining at control conditions (white bars) and in the presence of palmitate + high glucose (grey bars) during a 24h culture period.

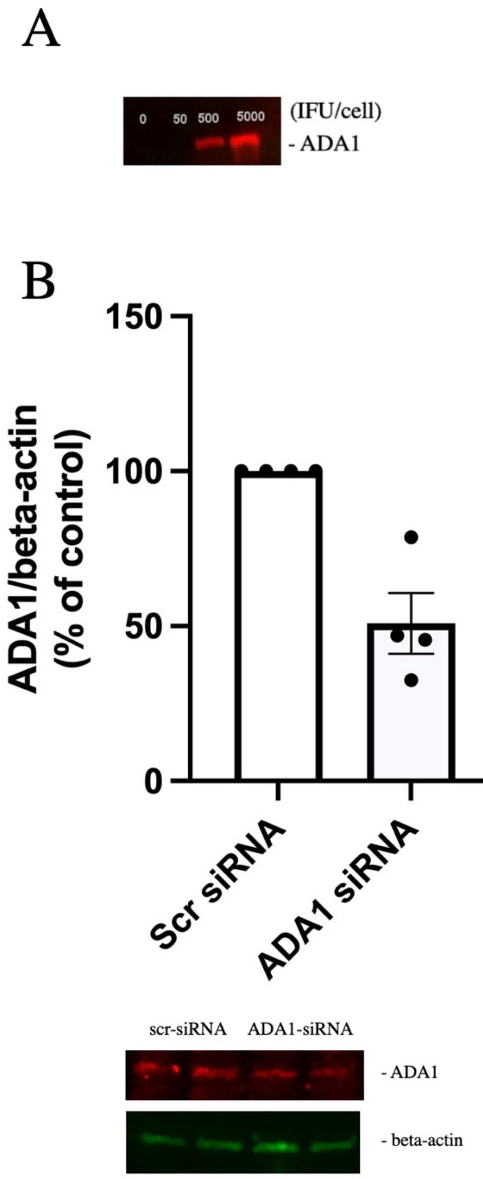

Suppl. Figure 5

(A) ADA1 adenoviral transduction results in increased ADA1 expression. EndoC-betaH1 cells were transduced with increasing concentrations of ADA1 adenoviral vectors and analyzed after 2 days by immunoblotting for ADA1 expression (A).

(B) (B) ADA1 siRNA reduces ADA1 protein expression.

EndoC-betaH1 cells were lipofected with scrambled and ADA1 specific siRNA and analyzed after 2 days by immunoblotting for ADA1 expression (B). ADA1 was expressed per beta-actin.

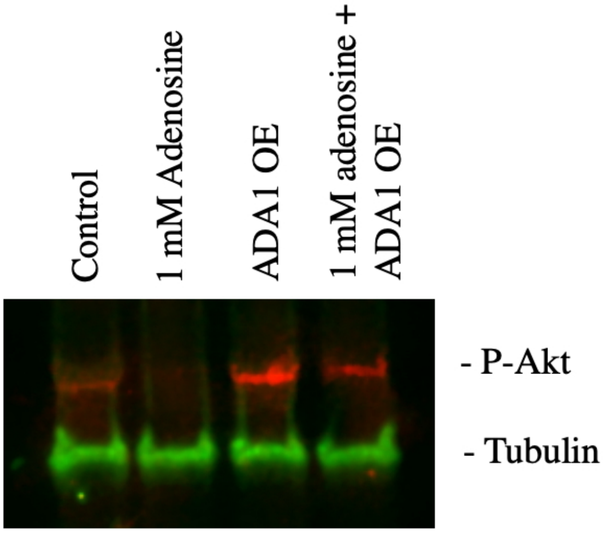

Suppl. Figure 6

ADA1 overexpression (OE) results in increased Akt signaling

Immunoblot analysis of Akt phosphorylation in EndoC-betaH1 cells exposed for 10 min to 1 mM adenosine. Cells were transduced with control or ADA1 adenoviral vectors 24h prior to the adenosine exposure. Results are representative for two independent experiments.

Target Detail	Target Rank	Target Score	miRNA Name	Gene Symbol
Details	1	94	hsa-miR-4533	submission
Details	2	85	hsa-miR-3663-3p	submission
Details	3	83	hsa-miR-30e-3p	submission
Details	4	83	hsa-miR-30d-3p	submission
Details	5	83	hsa-miR-30a-3p	submission
Details	6	82	hsa-miR-3064-3p	submission
Details	7	66	hsa-miR-6872-3p	submission
Details	8	62	hsa-miR-4715-3p	submission
Details	9	62	hsa-miR-4758-3p	submission
Details	10	61	hsa-miR-10b-3p	submission
Details	11	59	hsa-miR-4495	submission
Details	12	54	hsa-miR-1252-5p	submission
Details	13	51	hsa-miR-6815-3p	submission
Details	14	50	hsa-miR-6501-5p	submission

Suppl. Figure 7

Prediction of miRNAs that target the 3'-UTR of human ADA1 mRNA.

The prediction was obtained by using the "mirdb.org" site.
